# Supplementary material for: Epitaxial Hexagonal Boron Nitride for Hydrogen Generation by Radiolysis of Interfacial Water
Source: Nano Lett. 2023 Jan 23;23(4):1267–72. doi: 10.1021/acs.nanolett.2c04434 (PMC9951249; doi:10.1021/acs.nanolett.2c04434)
Supplement: Supplementary file 1 — nl2c04434_si_001.pdf [file nl2c04434_si_001.pdf]

Supporting Information for

Epitaxial hexagonal boron nitride for hydrogen generation by radiolysis of interfacial water

Johannes Binder,* Aleksandra Krystyna Dabrowska, Mateusz Tokarczyk, Katarzyna Ludwiczak, Rafal Bozek, Grzegorz Kowalski, Roman Stepniewski, and Andrzej Wysmolek

Faculty of Physics, University of Warsaw, ul. Pasteura 5, 02-093 Warsaw, Poland

E-mail: johannes.binder@fuw.edu.pl

Bubble formation in a scanning electron microscope

Videos are available online.

Bubble deformation during pressure cycles

A video (recorded in an optical microscope), corresponding to Figure 4 (b) in the main text, is available online.

Raman spectrum of epitaxial hBN grown by MOVPE

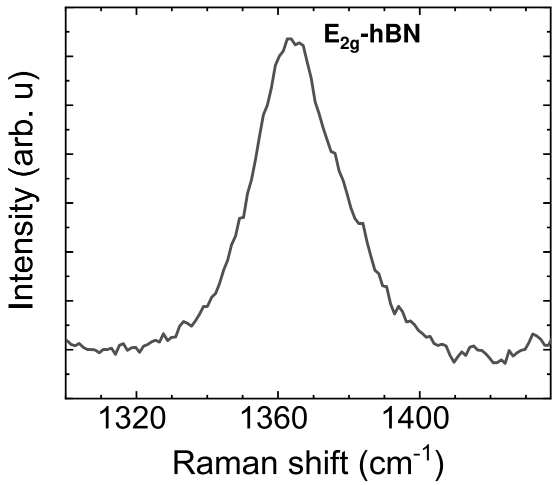

Figure S1: Raman spectrum showing the E_{2g}^{high} band of hBN. This band is a clear indication of sp^2-hybridized BN. A luminescence background has been subtracted.

X-ray diffraction of hBN

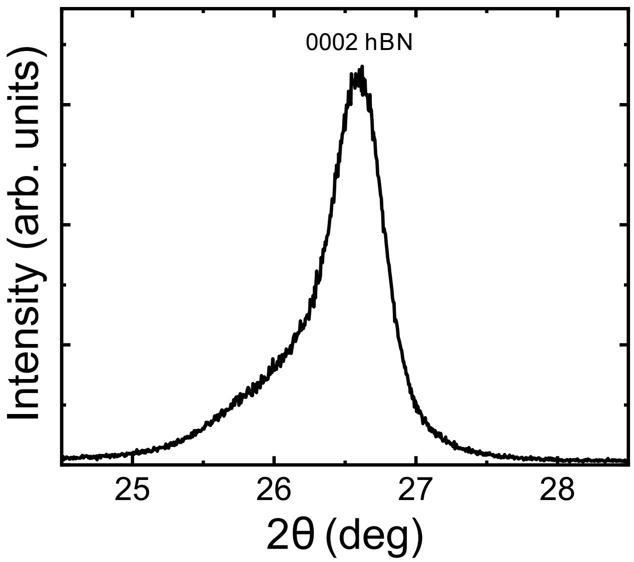

Figure S2: X-ray diffraction of MOVPE grown epitaxial hBN. A standard 2 theta/omega scan for the sample shown in Figure 1 in the main text. A prominent 0002 peak of hBN is observed. The asymmetric shoulder can be ascribed to a turbostratic component. A more detailed analysis can be found in ref[1]

References

(1) Dabrowska, A. K.; Tokarczyk, M.; Kowalski, G.; Binder, J.; Bozek, R.; Borysiuk, J.; Stepniewski, R.; Wysmolek, A. Two stage epitaxial growth of wafer-size multilayer h-BN by metal-organic vapor phase epitaxy – a homoepitaxial approach. *2D Materials* **2020**, *8*, 015017.
